# Pharmacological Overview of Galactogogues

**DOI:** 10.1155/2014/602894

**Published:** 2014-08-31

**Authors:** Felipe Penagos Tabares, Juliana V. Bedoya Jaramillo, Zulma Tatiana Ruiz-Cortés

**Affiliations:** Biogenesis Research Group, Agrarian Sciences Faculty, University of Antioquia, Medellin, Colombia

## Abstract

Galactogogues are substances used to induce, maintain, and increase milk production, both in human clinical conditions (like noninfectious agalactias and hypogalactias) and in massification of production in the animal dairy industry. This paper aims to report the state of the art on the possible mechanisms of action, effectiveness, and side effects of galactogogues, including potential uses in veterinary and human medicine. The knowledge gaps in veterinary clinical practice use of galactogogues, especially in the standardization of the lactogenic dose in some pure drugs and herbal preparations, are reviewed.

## 1. Introduction 

Milk production is essential for optimal feeding of infants and has a direct impact on growth, development, and health in neonatal period [1]. Breastfeeding is influenced by nutritional and nonnutritional factors (associated with endocrinology, health, climate, and management) that affect milk synthesis and secretion. These factors modulate physiological actions that regulate situations such as noninfectious agalactias and hypogalactias, the latest being the main problem of breastfeeding women [2]. Galactogogues are synthetic or plants molecules used to induce, maintain, and increase milk production [3], which mediate complex processes involving interaction between physical and physiological factors. Among the most important factors are hormones such as prolactin (PRL). However, somatotropine, cortisol, insulin, leptin, estrogen, progesterone and medroxyprogesterone [2], oxytocin, recombinant bovine somatotropin (rBST), and thyrotropin releasing hormone (TRH) also play important role as galactogogues (Table 1).

Most common galactogogues for human use are metoclopramide, domperidone, chlorpromazine, and sulpiride (Table 1); their remarkable side effects in mothers are xerostomia (dry mouth syndrome or hyposalivation), gastrointestinal disorders, cardiac arrhythmia, lethargy, sedation, extrapyramidal symptoms such as hypertension, tremor, tic, facial seborrhea, and hyperhidrosis, and even sudden death. In infants that ingested milk from treated mothers symptoms include intestinal discomfort, lethargy, and sedation [2]. The main galactogogue used in cattle is rBST which has reported adverse health effects that directly affect animal welfare [4, 5].

Plants with galactogogues components include fenugreek (*Trigonella graecum foecum*), fennel (*Foeniculum vulgare*), goat's rue (*Galega officinalis*), asparagus (*Asparagus racemosus*), anise (*Pimpinella anisum*), and milk thistle (*Silybum marianum*) [6, 7] (Table 2). Nowadays, herbal preparations are known to increase significantly milk production in women, goats, cows, and other species. This research area is very important for human breastfeeding medicine and in veterinary dairy industry [7–11].

There are numerous references about herbal medicine and breastfeeding. However, they are mainly based on empirical traditions and on human studies. This information could be deficient in systematization, is unstructured, heterogeneous, and thus has nonverifiable quality. From previously mentioned plants classified as galactogogues, there are currently available studies for efficacy and safety, but their mechanisms of action have not been elucidated yet [3, 12]. Publications generally focus on the effects with no emphasis on the mechanism by which milk production stimulation is achieved. The increased use of herbal medicine is also encouraged by a trend towards organic production, mainly in European markets, and the growing evidence on its safety and efficacy [13]. Several factors explain the tendency to use botanical galactogogues: adverse effects of synthetic drugs and a better understanding of chemistry, pharmacology and clinical use of botanical drugs and their derivatives, the development of analytical methods that facilitate quality control, and the development of new ways of preparation and administration [14, 15]. Many nutraceutical and phytopharmaceutical preparations are not approached in many countries; to develop and sale these preparations, it is necessary to have the basic knowledge of their chemical composition and of the mechanisms implicated on its galactogogue action. The following are also required: good agricultural practices (GAP), good laboratory practices (GLP), good manufacturing practices (GMP), and quality control standards to ensure the efficacy, safety, and composition of the products produced from these plants [16–18]. The use of herbal products in dairy industry relies on the new trend in dairy sector of organic dairy farming [19].

This paper reports and reviews potential uses of galactogogues in human and veterinary medicine, in both clinical uses and feeding practices of dairy animals, with emphasis on the possible mechanism of action relating drugs and plants knowledge, their efficacy and adverse effects. It also exposes gaps knowledge about galactogogues in veterinary clinical practice, especially in dose standardization of some pure drugs (with only one molecule in the pharmaceutical preparation) and herbal preparations.

## 2. Synthetic Galactogogues 

**Figure 1:**
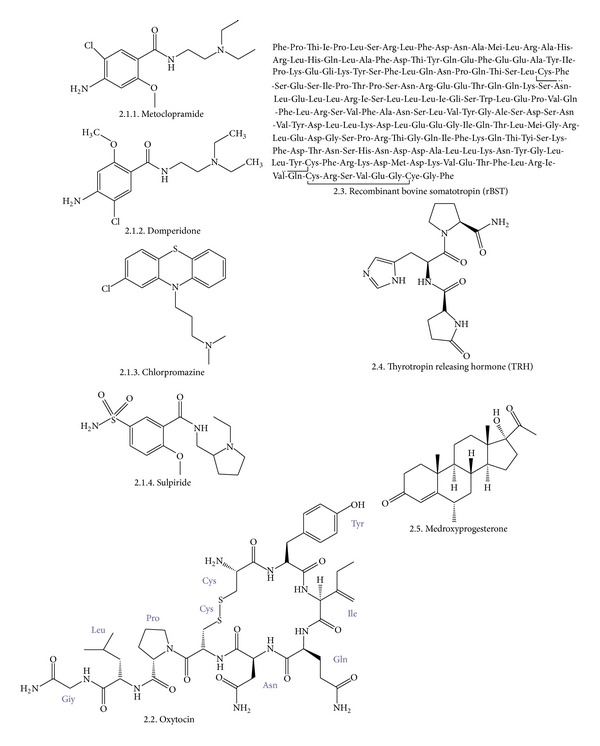
Synthetics galactogogue drugs structure.

Among synthetic molecules used to increase lactation, the dopamine antagonists, such as antiemetics metoclopramide and domperidone and such as antipsychotics sulpiride and chlorpromazine. Hormone synthetic analogs such as oxytocin, rBST, TRH, and medroxyprogesterone are also included in the synthetic galactogogues list [2].  Figure 1 depicts the basic structures of synthetic galactogogues mentioned in this review.

### 2.1. Dopamine Antagonists

These drugs block the dopamine 2 receptors (D2R) in the central nervous system which induces an increase of PRL synthesis in lactotrophic cells of the anterior pituitary [20–22]. Activation by an agonist of D2R, a G protein receptor, induces via subunit Gα0 the K^+^ channels opening, increases intracellular concentration of this ion, and reduces Ca^2+^ entry and its intracellular concentration. This effect is also induced by another pathway: inhibition of phospholipase C (PLC) and protein kinase C (PKC); reducing the Ca^2+^ mobilization from endoplasmic reticulum (ER), the low Ca^2+^ inhibits vesicle formation and PRL secretion. The activation of D2R also turns active the subunit Gαi, which inhibits adenylyl cyclase (AC), and decreases the concentration of adenine monophosphate (cAMP) [23], suppressing cAMP dependent protein kinase (PKA). Finally, inhibition of both kinases, PKC and PKA, inactivates PRL gene expression [24, 25].

**Figure 2:**
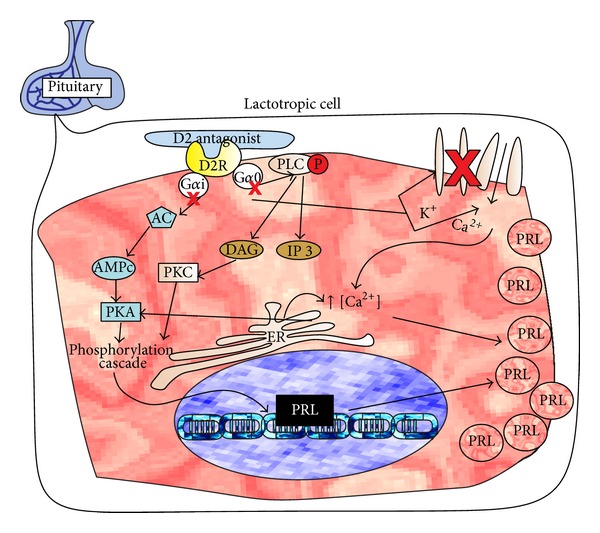
Proposed mechanism of action of dopamine 2 antagonists. In the pituitary gland, antagonists bind to the receptor (D2R) dopamine 2 and induce PRL gene expression, blood level of PRL increases, milk protein synthesis rate increases, and mammary epithelial cells (MEC) proliferation is stimulated.

When an antagonist binds to the receptor, those pathways are blocked, and the synthesis and release of PRL are activated. This high blood level of PRL increases milk protein synthesis rate and mammary epithelial cells (MEC) proliferation (Figure 2) [26].

#### 2.1.1. Metoclopramide

This drug was originally commercialized in Europe as an antipsychotic and later in the US as a gastrokinetic agent that increases gastrointestinal motility. Its first reported use as a galactogogue was in 1975 [27] and has been evaluated in many clinical trials [28]. In humans, adverse effects have been reported in mothers such as anxiety, and several gastrointestinal disorders, insomnia [2], severe depression, and seizures and in infants that consume milk from treated mothers cause intestinal discomfort [2]. Half-life reported in humans is 156.7 minutes [29] and its plasma half-life in dogs is about 90 minutes [30]. In humans, 10 mg administered by oral route (PO) three times a day during 10 days increases milk production [31]. It is used in small animal veterinary medicine to treat cases of secondary hypogalactia or agalactia at doses of 0.1-0.2 mg/kg subcutaneously (SC) every 6–8 hours for 4 to 6 days [32].

#### 2.1.2. Domperidone

Its first use as a galactogogue was reported in 1983 [33]. It was used to increase milk production in mothers of premature infants [34], but it was not approved by the Food and Drug Administration (FDA) in the US and domperidone use in human clinical trials has not been associated with adverse effects in infants, but in mothers it was associated with xerostomia, gastrointestinal disorders, cardiac arrhythmia, and sudden death, and this should be taken into account in veterinary practice [2]. There are recent human data where no maternal or neonatal adverse effects were reported [35]. The half-life reported in human is 7.5 hours [36]. Women enhance lactation with 10 mg of domperidone PO 3 times daily [37]. In dogs and cats, the domperidone medical use in secondary agalactia or hypogalactia is recommended at doses of 2.2 mg/kg SC, every 12 hours for 4–6 days [32]. In equine domperidone administered dose is 1.1 mg/kg PO every 12 hours to increase PRL blood concentration and milk production [38]. Domperidone is effective in preventing the signs of tall fescue toxicosis (including hypogalactia or agalactia) in horses without neuroleptic side effects [39].

#### 2.1.3. Chlorpromazine

Like in other neuroleptics, little is known about pharmacokinetics of chlorpromazine in mothers or infants during breastfeeding [40]. Chlorpromazine administered in doses of 15 mg/kg of body weight in rats during 5 days was effective in inducing lobuloalveolar growth and initiation of milk secretion initially primed with 10 *μ*g estradiol daily for 10 days [41]. Also, this neuroleptic increases milk production and weight gain in women with hypogalactia at doses of 25 mg, 3 times a day for a week [42]. The half-life reported in humans is 16–30 hours [43]. Short and long term use cause adverse effects in the development of the central nervous system (CNS) as documented by extrapyramidal symptoms in mothers and lethargy in infants that consumed milk. This could induce changes in CNS development in neonate because of alterations in the undeveloped brain [44]. The possible effects listed for the acepromazine use in animals are hypotension and contradictory effects such as CNS stimulation and bradycardia [30]. In felines chlorpromazine may cause extrapyramidal signs when used at high dosages. These can include tremors, shivering, rigidity, and loss of the righting reflexes. Lethargy, diarrhea, and loss of anal sphincter tone may also be seen [30]. In horses ataxic reactions with resultant excitation, panic reactions, and violent consequences may develop. These ataxic periods may cycle with periods of sedation. Because of this effect, chlorpromazine is rarely used in equine medicine today [30]. Animals in treatment with chlorpromazine should not be exposed to sun because it may induce phototoxic reactions [45, 46].

#### 2.1.4. Sulpiride

It was shown as a drug with galactogogue potential effect when increased serum PRL was observed in women [47]. Several clinical studies support its efficacy; one of these included 130 primiparous women: 66 treated with doses of 50 mg of oral sulpiride, every 12 hours during 7 days, and 64 as placebo group. The treatment resulted in an increase in PRL serum levels as in milk secretion [48]. A previous study reported an effective oral sulpiride dose of 50 mg every 8 hours for 4 weeks in women with hypogalactia; in this investigation serum PRL concentrations increased during the first 2 weeks, while the control group decreased and infants of treated mothers showed higher weight gain than those of the placebo group after 28 days of sulpiride treatment [49]. These results were confirmed by other studies [48, 50]. Plasma half-life of sulpiride in dogs was 1.6–3.4 hours [51, 52] and in humans was 7.15 hours [53]. Adverse effects reported in women were headache, fatigue, extrapyramidal symptoms, acute dystonic reactions, and endocrine disruption [2, 50]. In equine, sulpiride used at dose of 1.1 mg/kg PO twice a day [54] and 0.5 mg/kg intramuscularly (IM) twice a day increased PRL blood concentration and milk production [38].

### 2.2. Oxytocin (OT)

**Figure 3:**
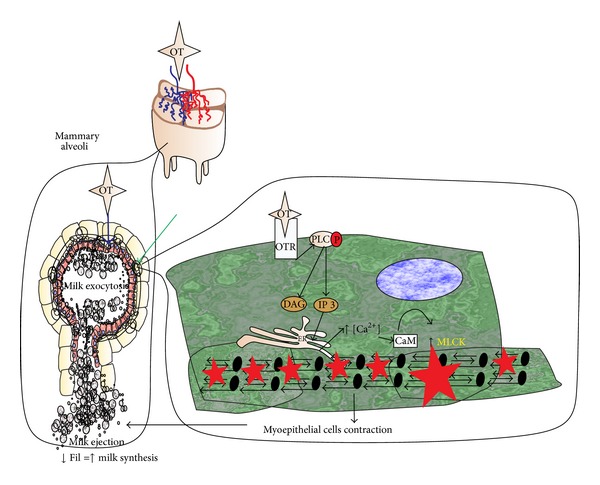
Proposed mechanism of action of oxytocin (OT). This hormone induces contraction of the myoepithelial cells (green arrow), via a G protein receptor. OT also induces exocytosis of milk in MEC (blue arrow) by intracellular Ca^2+^ increased pathways. Myoepithelial cells contraction and MEC exocytosis induce milk ejection; the continued milk ejection results in a decrease of a protein milk synthesis reversible blocker: feedback inhibitor of lactation (FIL), and this milk and FIL removal of mammary gland will promote then the milk synthesis.

The major sites of expression of this peptide hormone are located in the magnocellular neuron region in the supraoptic and paraventricular hypothalamic nuclei [55]. It has been used to induce milk ejection in cases where dysfunction has been associated with this reflex [56]. This hormone induces contraction of myoepithelial cells via G protein receptor, and PLC is activated and induces the formation of diacylglycerol (DAG) and inositol 1,4,5-triphosphate (IP3), by hydrolysis of membrane lipid phosphatidylinositol 4, 5-bisphosphate (IPI2). The IP3 induces intracellular Ca^2+^ release, and this active Ca^2+^-calmodulin system triggers the activation of myosin light-chain kinase (MLCK) which initiates smooth muscle contraction in mammary myoepithelial cells [55] (Figure 3). In rabbit, OT not only stimulates milk ejection by the contraction of mammary myoepithelial cells, but also induces exocytosis of milk synthesis in the MEC [57]. With effects in myoepithelial and MEC, OT induces milk ejection and this milk removal also removes feedback inhibitor of lactation (FIL), a milk glycoprotein that induces reversible block of protein synthesis of the MEC. Thus, reduction of FIL induces milk synthesis [58] (Figure 3). OT can increase milk production and is indicated in agalactia or hypogalactia for dysfunction of milk ejection reflex in stress or premature birth cases [56]; it is also used in mastitis treatment [59, 60]. The half-life reported in goats is 22 minutes [61]; in pigs, 127 seconds [62]; in rats, 1.46 minutes [63]; in cows, two half-life data were reported: 7–9 minutes and 25 minutes [64, 65]; in equines it was determined to be 6.8 minutes [66]; in humans, 272 seconds [67]. There are no reports about OT adverse effects in women or infants [2, 68]. When used appropriately at reasonable dosages, oxytocin rarely causes significant adverse reactions [30]. Most adverse effects are a result of using the drug in inappropriate individuals (adequate physical exam and monitoring of patient are essential) or at too high doses [30]. Most of the older dosage recommendations for dogs or cats are obsolete as minidoses have been found to improve the frequency of uterine contractility and are less hazardous to the bitch (uterine rupture) and to the fetuses (placental compromise) [30]. Repeated bolus injections of oxytocin may cause uterine cramping and discomfort [30]. The use of oxytocin in dairy animal as galactogogue is banned in India and other countries because its continuous use in each milking affects the animal welfare [69]. In dogs and cats reported medicated doses are 0.5–2.0 IU/kg dose SC every two hours [32]. In bovine SC injection dose of 20 IU per animal at each milking throughout lactation increased milk production [70]. The doses mostly used in goat and sheep are 1–5 IU SC every milking [71]. In swine reported doses are between 0.025 and 0.05 IU in intravenously (IV) rapid injection every milking [72]. Equine reported IM dose 20 IU per animal every milking [73].

### 2.3. Recombinant Bovine Somatotropin (rBST)

The rBST approved in dairy cows is the 190-amino-acid variant with leucine at position 127, and it has an extra methionine at the NH2 terminus [5, 74] (Figure 1). In 1979, rBST was developed in bioreactors (an* E. coli* strain); three years later its* in vivo* galactagogue action was published [75]. Its use was approved in US in 1993 and commercialized one year later. The rBST increases milk production approximately 2.25 to 6.6 liters/cow/day and increases lactancy in 30 to 100 days [5, 76]. In 1998 more than 100 million doses of rBST were sold around the world and it is estimated that in 1999 about 30% of 9 million dairy cows in the US were treated with this drug. Cows were treated with 500 mg SC every 14 days throughout the lactation period and maximum increase in production is achieved after third or fourth injection [5].

**Figure 4:**
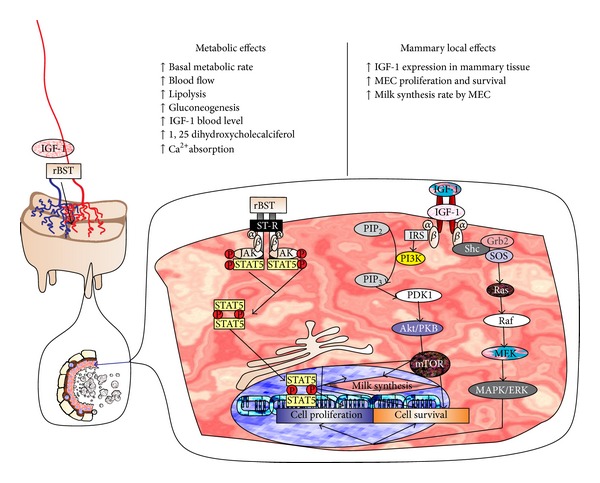
Proposed mechanism of action of recombinant bovine somatotropin (rBST). This hormone has direct effects on basal metabolic rate and breast parenchyma; the effects on the MEC (blue arrow) are mediated by rBST/ST-R complex, which stimulates JAK2/STAT5 pathway and by IGF-1R/IGF-1 which promotes and upregulates IRS/PI3K/(AKT/PKB)/mTOR and Ras/Raf/(MAPK/ERK) pathways. This will induce cell proliferation and survival and increase milk protein synthesis in MEC.

This hormone has direct effects on breast parenchyma and basal metabolic rate. This promotes increases in milk synthesis, blood flow, and viability of MEC, along with increases in insulin-like growth factor 1 (IGF-1) protein in liver and mammary tissues [77, 78]. Other effects were observed on lipolysis, gluconeogenesis, and production of 1,25 dihydroxycholecalciferol and Ca^2+^ absorption [73, 78]. The effects on mammary epithelium are mediated by stimulation of somatotropine receptor (ST-R), which in synergy with the PRL pathway stimulates the Janus kinase/signal transducer and activator of transcription 5 (STAT5), the main lactogenic mediator of MEC proliferation, survival, and milk gene expression signaling [79–81]. Activation of the IGF-IR occurs following IGF-I binding to the α-subunit of the IGF-IR on epithelial cells, leading to autophosphorylation of the *β*-subunit by an intrinsic tyrosine kinase. These events can lead to the activation of a number of downstream [82, 83] pathways including the insulin receptor substrate (IRS) phosphorylation, which are involved in the upregulation phosphorylation of the phosphatidylinositol 4,5-bisphosphate (PIP_2_) to phosphatidylinositol (3,4,5)-triphosphate (PIP_3_) by phosphatidylinositol 3-kinase (PI3K); the PIP_3_ increment is followed by phosphoinositide dependent kinase-1 (PDK1) [84], serine/threonine kinasealso known as protein kinase B (Akt/PKB), and mammalian target of rapamycin (mTOR) activation that induces MEC proliferation, survival (antiapoptotic), and milk synthesis gene expression [83, 85–87]. Another IGF-1 activated pathway is the rat sarcoma protein [88]/rapidly accelerated Fibrosarcoma kinase (Raf)/mitogen activated protein kinase (MAPK) (also known as Ras-Raf-mitogen-activated protein kinase kinase (MEK)-ERK pathway), which, after the binding of IGF-1 to its receptor, induces phosphorylation of tyrosine residues, docking protein such as growth factor receptor-bound protein 2 (GRB2). This factor contains an Src homology 2 domain (Shc) that binds to the phosphotyrosine residues of the activated receptor GRB2 and binds to Son of Sevenless (SOS) to produce GRB2-SOS complex and docks to phosphorylated IGFR, SOS becomes activated and then induces Ras activation, Ras activates Raf, and this induces a phosphorylation cascade that activates MEK and mitogen-activated protein kinase also known as extracellular signal-reduced kinases MAPK/ERK [89–91]. Both IRS/PI3K/AKT(PKB)/mTOR pathway and Ras/Raf/(MAPK/ERK) pathway activated by IGF-1 and the JAK/STAT5 pathway activated by rBST/ST-R induce MEC proliferation and survival and increase milk protein synthesis, finally explaining the galactogogue actions of rBST [92] (Figure 4). Its half-life in Holstein cows is 54.8 minutes [93]. Somatotropin is the main galactogogue used in cattle. However, its use not only results in gain in productive efficiency and profitability but has also generated ethical dilemmas, in terms of animal welfare and health and potential risks for consumers. Contraindications in cattle include low pregnancy rates, increased open days [94], increased incidences of retained placenta [95], clinical and subclinical mastitis [96, 97], laminitis, digestive disorders, reduced feed intake, allergic reactions [4], and decreased hemoglobin and hematocrit [98]. The FDA reports that between 1994 and 2005 they received about 2408 cases of adverse reactions to this treatment [4]. These facts triggered the decision of the European Union members, Canada, and other countries to prohibit its administration [4, 5].

### 2.4. Thyrotropin Releasing Hormone (TRH)

**Figure 5:**
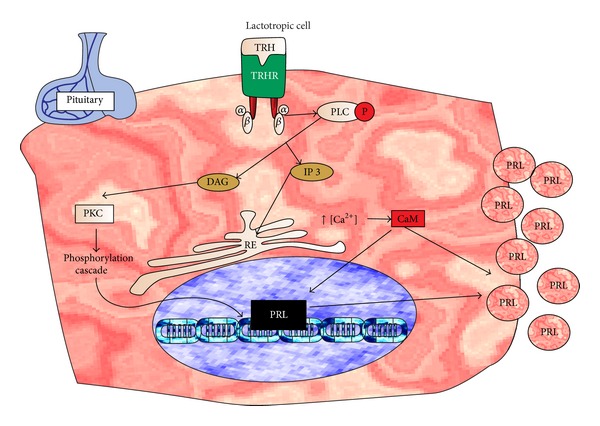
Proposed mechanism of action of thyrotropin releasing hormone (TRH). The TRH molecule binds to its receptor in lactotrophic cells of the pituitary gland and stimulates Ca^2+^/CaM release, which induces the PRL gene expression. Furthermore, the increase in intracellular CA^2+^/CaM stimulates the release of PRL stored in vesicles. This will increase PRL blood levels and promotes more milk synthesis.

This peptide hormone is synthesized in the hypothalamus, stimulating the secretion of thyroid stimulating hormone (TSH) and PRL by the anterior pituitary [99–101]. TRH is the principal physiological factor stimulating the fast release of PRL [99, 102]. Synthetic TRH applied IV can significantly increase serum PRL in proestrous female and in normal and estrogen-primed male rats, 10 min after injection [103]. Subcutaneous administration of TRH was also effective to increase plasma PRL levels in lactating cows [104]. Women treated with synthetic TRH 20 mg PO three times a day had high blood concentrations of PRL [105]. In another study, TRH administration for one month, at doses of 5 mg twice a day PO, did not change PRL blood concentration in human [106]. TRH has been effective in the induction of lactation in mothers with agalactia 10–150 days after birth [107], but its galactogogue effect is variable [108]. Its half-life in rats was found to be 4.16 minutes [109]. The TRH molecule binds to its receptor in the lactotrophic cells triggering the activation of PLC and increasing the formation of diacylglycerol (DAG) and inositol 1,4,5-triphosphate (IP3). DAG activates protein kinase C (PKC) and PKC promotes phosphorylation pathways that culminate in PRL gene expression; IP3 induces release of Ca^2+^ from endoplasmic reticulum, forming the complex Ca^2+^-calmodulin (CaM) [110], and this complex induces the PRL gene expression [111, 112]. Furthermore, the increase of intracellular CA^2+^ and CaM stimulates the release of the PRL stored in vesicles [112, 113] (Figure 5).

About its elimination in milk, no data are available [2]. TRH administration increases maternal plasma levels of thyroxine T4 and triiodothyronine T3; however, both hormones appear in low concentrations in milk [99]. No side effects have been found in infants [105]. Some cases of iatrogenic hyperthyroidism and brief episodes of sweating have been reported in mothers [106, 108]. There are no clinical studies about its use in veterinary medicine, and more research is needed.

### 2.5. Medroxyprogesterone

It is a steroidal synthetic progesterone (a progestin). This drug causes hyperplasia of mammary secretory epithelium in macaques [114] and mice, with its activity being associated with epidermal growth factor (EGF) [115]. However, there are limited clinical studies in women suggesting that this drug is effective in increasing serum PRL and milk production [116–118]. Medroxyprogesterone acetate biological half-life in human is 40–60 hrs [119]. In human, medroxyprogesterone was found in plasma and in milk at a 1 : 1 ratio [120]. No adverse effects were reported in infants and in mothers amenorrhea was described [121]. Reported effective dose in humans is 150 mg IM every 3 months, beginning at week 2 postpartum and repeating at week 14 [122]. It is considered compatible with breastfeeding [121] and its mechanism of action is not well known [2].

## 3. Herbal Galactogogues 

Some plants have been used in many cultures to stimulate milk production in women and in dairy animals [123]. Galactogogue effect of various plants has been studied and there is evidence that milk synthesis can be increased and that most of them are safe in humans [40], cows [7, 88, 124, 125], goats [126–129], and buffaloes [130–132]. Several herbal galactogogues have been reported as safe substances that in appropriate and economic doses can be used therapeutically in domestic animals [67] and in food supplements of dairy herds [7, 126]. The herbal derivative products use in dairy industry relies on the new trend in dairy sector of organic dairy farming [19, 133]. Some herbs demonstrate efficacy in increasing milk production in women with mammary hypoplasia [134].

However, pharmacokinetics and pharmacodynamics of active ingredients present in galactogogues plants are not well characterized and further research is compulsory to determine their mechanisms of action and to establish therapeutic ranges, dosage, and possible side effects in different domestic species and humans [123]. Some clinical trial results have shown several limitations including small sample size, insufficient randomization methods, poorly defined eligibility criteria, use of poly-herbal interventions, and variable breastfeeding practices among enrolled subjects [3]. Plant drugs, also known as herbal drugs, phytopharmaceuticals, or phytomedicines are plant-derived medicines that contain a chemical compound or more usually mixtures of chemical compounds that act individually or in combination on the animal body to prevent disorders and to restore or maintain health [16] or to improve the animal production [135].

Crude plants, herbal teas, decoction, and alcoholic extracts are also traditional ways of using medicinal plants. Very often these plant materials are used in a nonstandardized manner [16]. However, nowadays more and more emphasis is being put on the use of standardized materials and preparations to ensure the efficacy, safety, and composition; this is called as pharmaceutical quality [16–18]. To develop and sale these preparations studies of phytochemical composition, pharmacodynamic and pharmacokinetic are necessary, but also good agricultural practices (GAP), good laboratory practices (GLP), good manufacturing practices (GMP), and quality control standards are required [16–18]. The complementarity of analytical methods like high performance liquid chromatography (HPLC) and gas chromatography** (**GC) is of paramount importance for analyzing both the lead and the minor compounds [16]. For the pharmaceutical quality level also it is important to make assessment of microbial contamination, raw materials adulteration, and side-effects (toxicity) of plant extracts [16, 136].

**Figure 6:**
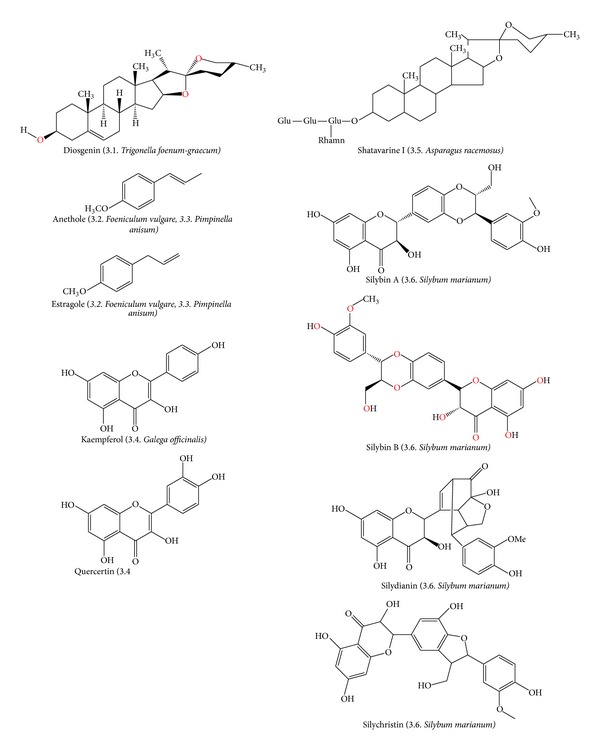
Phytoestrogenic molecules present in some botanical galactogogues.

**Figure 7:**
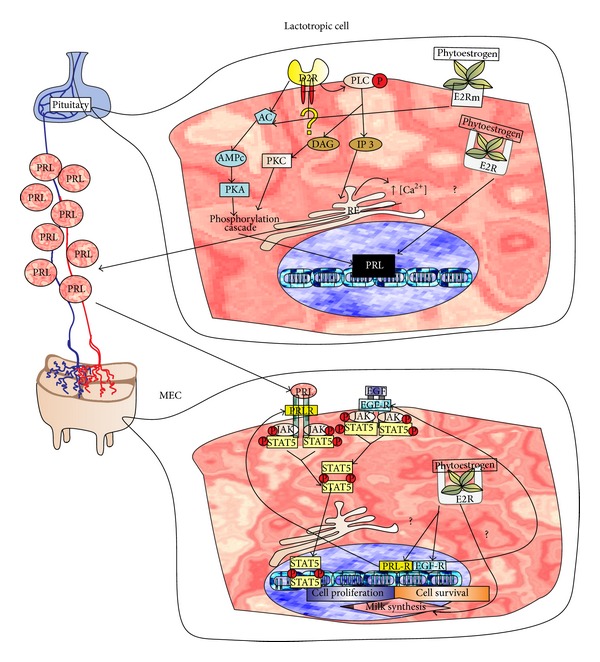
Proposed galactogogue mechanism of action of phytoestrogen molecules in anterior pituitary and mammary gland. The E2-like action may induce PRL expression in anterior pituitary lactotrophic cells and milk production in MEC by indeterminate pathways (?). In lactotrophic cells, upregulation of PRL gene expression and secretion occurs directly by E2R and indirectly by mE2R inducing D2R inhibition. In MEC, PRL-R and EGF-R expressions are induced and milk synthesis, cell proliferation, and survival gene expression are suggested.

It is necessary to develop well-designed and well-conducted clinical trials that address the above limitations to generate strong evidence of the efficacy and safety, as a basis for producing herbal galactogogues preparations [3, 137]. The pharmacological research of botanical galactogogues should study nutritional values (macro- and micronutrients) as wells as therapeutic potentials (secondary metabolites and their activities) [138]. Turkyilmaz et al. suggested that the herbal galactogogues effect could be mediated by phytoestrogenic action [139] and that some molecules may have effects similar to 17*β*-estradiol (E2), an endogenous estrogen that promotes the proliferation of MEC [140]. The supply of genistein (isoflavone phytoestrogen) induces mammary gland hyperplasia in sows [141].  Figure 6 depicts the chemical structures of phytoestrogens that are mentioned in this review. We hypothesize that if the phytoestrogen molecules have E2-like action, these molecules could induce the expression of PRL receptor (PRLR) [142] and EGF receptor (EGFR) [143] and could upregulate casein production and lactose synthetase activity in MEC [144]. E2 triggers PRL gene expression through at least two independent and undetermined pathways in pituitary lactotropic cells. A first route is characterized to act through the intracellular receptor E2 (E2R) that finally increases levels of PRL [145, 146] and increases secretion of milk. These effects are mediated by the pathway triggered by α isoform of the membrane-associated estrogen receptor (mE2R) (Figure 7). The second route inhibits the pathway activated by D2R dopamine receptor, stimulating PRL production and proliferation of lactotrophic cells by increasing cAMP ending in PKA phosphorylation pathways that trigger PRL gene expression [107] (Figure 7). The following sections will review information about commonly used galactogogues plants.

### 3.1. *Trigonella foenum graecum* (Fenugreek/fenugreek)

Itis the most used commonly herbal galactogogue [3, 147]. It is a member of the Leguminosaefamily that is cultivated in many parts of the world, particularly in India, Mediterranean countries, north Africa, and southern Europe [123]. Reports indicate that seeds have mastogenic effect, stimulating growth of mammary gland [138]. This plant is used around the world as galactagogue in women due to its phytoestrogens' significant levels [148]. One study using* in vitro* assays found that fenugreek seeds contain estrogen-like compounds and that they stimulate pS2 (estrogen-induced protein) expression in a breast cancer cell line Michigan Cancer Foundation-7 (MCF-7); pS2 is frequently used as a marker for assessing the estrogenicity of a compound [148]. Phytoestrogens as diosgenin, a type of steroidal sapogenin, could explain the observed milk flow increase [3].

Recently, it was found that fenugreek induces a significant increase in milk production in women and decrease the time of neonates recovery weight [139]. Despite its widespread use, there is little research conducted on its pharmacodynamic and pharmacokinetics properties to determine the extent to which its components are excreted in milk.

Moreover, this plant has been shown to influence the maintenance of lactation in ruminants; buffaloes fed with seeds increase milk production, but it has not been clearly demonstrated whether its composition is altered [130]. In goats, it has been reported that feeding with 10 g daily of fenugreek seed increases milk production [127]. Attempting to elucidate the mechanism by which this plant increases milk production, it is proposed that the galactogenic effect could be mediated through increased feed intake in buffaloes [131].

Other studies suggest that stimulation of endogenous hormones secretion may be the way by which fenugreek exerts its action on increasing milk production. In goats feeding with fenugreek increased milk production and this effect might be mediated via PRL stimulation, because PRL concentrations were found to be significantly higher in the fenugreek fed goats compared to control group [149]. Similarly, in a recent study performed in goats, an increase of 13% in milk production was paralleled to an increase in serum somatotropine [128]. It is suggested also that plasma growth hormone in buffaloes could be candidate in mediating fenugreek action [131].

### 3.2. *Foeniculum vulgare* (Fennel)

It is the only species in the genus* Foeniculum*, found in temperate zones around the world, and it is a perennial and aromatic plant native of southern Europe, especially the Mediterranean coast, where it is considered as a wild herb [150]. The first report of its galactagogue properties was by a Greek botanist Pedanius Dioscorides (40–90 A.D). This plant may increase milk production and milk fat content in goats [151]. It has been used as a galactogogue in humans and no adverse effects have been reported yet [6, 123], in mice [152] or goats [136].* F. vulgare* has been used as an estrogenic agent for centuries. It has been reported to increase milk secretion, improve the reproductive cyclicity, facilitate birth, and increase libido [150]. It contains E2-like molecules, such as anethole and estragole [153, 154] (Figure 6).

### 3.3. *Pimpinella anisum* (Anise)

This herbal galactogogue is part of the Apiaceae family, a plant found in West Asia, Eastern Mediterranean, Mexico, and Spain. The main oil constituents, obtained from dried fruits, are trans-anethole (93,9%) and estragole (2,4%), which are pharmaceutical compounds that possess strong estrogenic activity which justifies its use as a galactagogue [7, 123, 155]. Aqueous and ethanolic extracts of* P. anisum* seeds can increase milk production in rats [156]. The aqueous (1 g/kg) and ethanolic extracts (1 g/kg) increased rats milk production significantly in about 68.1% and 81%, respectively, compared to the control group [156].

### 3.4. *Galega officinalis* (Goat's Rue)

It is an herbaceous plant from central and southern Europe. Its lactogenic value has to be considered according to reported increase in milk yield and lactation persistency when included in a daily diet in cows [88, 124, 129, 157] and sheep [158]. However, several members of the genus* Galega* have been listed as poisonous to livestock in New Zealand and USA [158, 159]. Genus* Galega* is considered to be of low palatability and high toxicity [159]; this latter due to high concentrations of guanidine derived molecules hidroxigalegin and galegin [160]. The toxic effects of* G. officinalis* in sheep may vary among individuals, but in all cases, doses over 5 g/kg are toxic [161]. In contrast with these reports of toxicity, lactogenic properties of* G. officinalis* were confirmed in sheep at daily doses of 2 g of dry matter/kg body weight from the first month after lambing and during 60 days; the result was a 16.9% increase in total milk yield, without any signs of toxicity [158]. In cows any adverse effects were reported in a diet of 25% concentrate feeds and 75%* G. officinalis*, with* ad libitum* intake [132]. In this regard, the administration of phytoestrogens in low doses or foods containing them could promote activation of some estrogen receptors in the animal and increase milk production. Several phytoestrogens have been isolated from methanol extracts of Goat's rue such as flavonol triglycosides, kaempferol, and quercetin [158, 162].

### 3.5. *Asparagus racemosus* (Shatavari)

This plant belongs to the Asparagaceaefamily and has its origins in India; its role as a milk production enhancing substance has been mentioned in several ancient Ayurvedic text books such as* Charaka Samhita* and* Susruta Samhita* [163]. It has phytoestrogenic properties [163] and it has been observed to increase milk secretion following administration of* A. racemosus* as Ricalex tablets in women suffering from hypogalactia [164]; the gradual decrease in milk secretion, on withdrawal of the drug, suggested that the increase in milk secretion was due to drug therapy only and not to any psychological effect [165]. In 2011, the root powder oral administration in women in a double-blind randomized clinical trial has demonstrated a threefold increase in PRL level in subjects of the research group compared to the control group [166]. However, in previous works authors did not observe any increase in PRL levels in* A. racemosus* treated females suffering from a secondary lactational failure [163]. In rats supplemented with the plant (2% of their diet), a lactogenic effect was reported [167]. Systemic administration of alcohol extract of* A. racemosus* in weaning rats increased weight of mammary glands, inhibited involution of lobuloalveolar tissue, and maintained milk secretion [168]. Other studies with alcohol extract of Shatavari demonstrated estrogenic effects in genital organs and in mammary glands in rats with hyperplasia in alveolar tissues and acini and with increased milk production [163]. A significant increase in milk yield has also been observed in pigs and goats after feeding with lactare (commercial herbal galactogogues with* A. racemosus* in its formulation) which also increased growth of the mammary glands, alveolar tissues, and acini [169]. Roots of* A. racemosus* also have shown galactogogue effect in buffaloes [170]. In rats, its methanolic roots extract in a dose of 100 mg/Kg/day for 60 days showed teratological disorders in terms of increased fetuses resorption, malformations as legs swelling, and intrauterine growth retardation with a small placental size [171]. Chemical analysis of Shatavari roots reveals the presence of steroidal saponins (as Shatavarins I-IV). Shatavarin I is the most glycosided molecule with 3 glucose and a rhamnose moieties attached to sarsasapogenin [172]; one hypothesis states that the estrogenic activity results from the hormone-like actions of these steroidal saponins [163, 166]. Another hypothesis declares that the growth of mammary tissue is caused by the action of released corticoids or PRL [163, 165, 166]. Although estrogens have a stimulating effect on the ductal epithelial cells, causing them to lengthen, their primary role seems to be the potentiation of PRL production [3].

### 3.6. *Silybum marianum* (Milk Thistle)

This medicinal plant has been used from ancient times by Theophrastus (4th century BC) who was probably the first to describe it under the name of “Pternix,” and later it was mentioned by Dioskurides in his “Materia medica” and by Plinius (1st century AD) [173]. Silymarin is a mixture of flavonoids extracted from seeds of this plant, which contains silybin, silydianin, and silychristin; molecules that show estrogenic effect in ovariectomized rats [174] and its major component, silibinin, bind to cytosolic estrogen receptors [175]. Human and animal studies suggest that milk thistle has promising lactogenic properties. In a study, after treatment with 10 g silymarin/cow/days PO in the peripartum (from 10 day sbefore calving to 15 days after calving), an increase in milk production of 5-6 L/day per cow was observed [176]. It is thought that the administration of silymarin after calf delivery improves physiological status of the cow, which leads to faster recovery, increased feed intake, and increased milk production. This finding was supported by the observation of reduction of blood *β*-hydroxybutyric acid and decreased outcomes of ketonuria in cows treated with silymarin [164, 176]. Silymarin elimination half-life in humans averages 6 hours [30]. Silymarin is apparently well tolerated when administered orally. In humans, GI disturbances have been reported on occasion (nausea to diarrhea). Patients who have allergies to other members of the* Asteraceae/Compositae* plant family (includes ragweed, marigolds, daisies, etc.) may exhibit allergic reactions to Milk Thistle derivatives [30]. In women orally treated for 63 days with 420 mg/day of silymarin a clear galactogogue effect was evident with an increase of 85.94% of daily milk production compared to 32.09% of the placebo group [154]. Female rats treated for 14 days with 25–200 mg/kg orally increased, in a dose dependent manner, the serum PRL levels [177]. It is known that silymarin elicited partial ER activation and silybin B were probably responsible for a majority of the weak ER-mediated activities of silymarin, whereas, its diastereomer, silybin A, was found to be inactive [173].

## 4. General Conclusions and Research Needs

Galactogogues, both synthetics and herbal, have been poorly studied in veterinary medicine. Most of the information about the effectiveness and safety of these substances as galactogogues was obtained by research in human; these studies were included in the review as a relevant comparative element, which are the basis for developing applications in veterinary and livestock practice, especially in massive dairy production. Nowadays, limited pharmacological knowledge exists about botanical galactogogues. The mechanisms of action and relevant pharmacological data were reviewed and hypotheses about its mechanism of action are postulated.* In vitro* studies in mammary secretory epithelial and lactotrophic cells are considered as reference models for pharmacological essays and determination of galactogogues action mechanisms and pathways; its limitations in terms of pharmacokinetic processes and systemic metabolic effects study in* in vitro* models are, however, recognized. Because of limited literature on this topic in veterinary practices, it is of interest to characterize the doses, characterization of phytochemical composition (molecules), formulations, and mechanisms of action, side effects, and drug interactions of galactogogues, mainly the herbals ones. This is an innovative research area that could be projected as sustainable strategies for massification and optimization of milk production in the dairy and swine industry (e.g., increasing weaning weight). These plants could be given as feed rations or its concentrated extracts (essential oils, alcohol extracts, lyophilized extract, among others) as supplements. Apparently, they are compatible with animal welfare but further basic and applied research about this issue is proposed.

## Figures and Tables

**Table 1:** Pharmacological overview of some galactogogues synthetic drugs.

Galactogogue drug	Proposed mechanism	Doses and dosage form	Adverse effects	Half-life
Metoclopramide	D2R antagonist, increase PRL secretion [2].	Human: 10 mg PO TID [31]. Canine/feline: 0.1-0.2 mg/kg, SC TID [32].	Humans: several gastrointestinal disorders, insomnia severe depression, and seizures and in infants that consume milk from treated mothers causing intestinal discomfort [2].	Humans: 156.7 minutes [29]. Dogs: 90 minutes [30].

Domperidone	D2R antagonist, increase PRL secretion [2].	Human: 10 mg PO TID [37]. Canine/feline: 2.2 mg/kg SC, every 12 hours for 4–6 days [36]. Equine: 1.1 mg/kg PO BID [38].	Humans: xerostomia, gastrointestinal disorders, cardiac arrhythmia, and sudden death [2].	Humans: 7.5 hours [36].

Chlorpromazine	D2R antagonist, increase PRL secretion [2].	Human: 25 mg PO TID [42]. Rat: 15 mg/kg [41].	Humans: extrapyramidal symptoms in mothers and lethargy in infants [2, 40]. Feline: in high doses tremors, shivering, rigidity, and loss of the righting, reflexes, lethargy, diarrhea, and loss of anal sphincter tone [30]. Equine: ataxic reactions, excitation, panic reactions and violent consequences, sedation [30], and phototoxic reactions [45, 46].	Humans: 16–30 hours [43].

Sulpiride	D2R antagonist, increase PRL secretion [2].	Human: 50 mg PO TID [49]. Equine: 1.1 mg/kg PO BID [54] or 0.5 BID IM [38].	Humans: headache, fatigue, extrapyramidal symptoms, acute dystonic reactions, and endocrine disruption [2, 50].	Dogs: 1.6–3.4 hours [51]. Humans: 7.15 hours [53].

Oxytocin	Induce milk ejection and this promotes milk synthesis by FIL decrease [28].	Canine and feline: 0.5–2.0 IU/kg dose SC EM [32]. Bovine: 20 IU SC EM [70]. Sheep and goat: 1–5 IU EM [71]. Swine: 0.025 and 0.05 IU IV EM [72]. Equine: 20 UI IM EM [73].	When used appropriately at reasonable dosages, oxytocin rarely causes significant adverse reactions [30]. Result of using the drug in inappropriate individuals (adequate physical exam and monitoring of patient are essential) or at too high doses [26]. In high doses they may cause discomfort, uterine cramping, hazardous of uterine rupture, and fetuses-placental compromise. [30].	Goat: 22 minutes [61]. Swine: 127 seconds [62]. Rat: 1.46 minutes [63]. Bovine: 7–9 minutes and 25 minutes [64, 65]. Equine: 6.8 minutes [66]. Humans: 272 seconds [67].

Recombinant bovine somatotropin (rBST)	Increase basal metabolic rate and nutrients bioavailability, in mammary gland Increase MEC proliferation, survival and milk synthesis [92].	Bovine: 500 mg SC every 14 days [5].	Bovine: low pregnancy rates, increased open days [94], increase incidences of retained placenta [95], clinical and subclinical, reduced food intake, allergic reactions, laminitis digestive disorders [5], decreased hemoglobin and hematocrit [98], and mastitis [96, 97].	Bovine: 54.8 minutes [93].

Thyrotropin releasing hormone (TRH)	Increase PRL blood levels [102].	Humans: 20 mg PO TID [105].	Human: hyperthyroidism and brief episodes of sweating [106, 108].	Rat: 4.16 minutes [109].

Medroxyprogesterone	Not reported	Humans: 150 mg IM every 3 months [122].	Human: amenorrhea [121].	Human: 40–60 hrs [119].

Abbreviations: BID, twice daily; TID, three times daily; PO, oral administration; SC, subcutaneous; IM, intramuscular; EM, every milking.

**Table 2:** Pharmacological overview of some botanical galactogogues.

Herbal galactogogue	Proposed mechanism	Common doses and dosage form	Adverse Effects	Half-life
Fenugreek (*Trigonella graecum foenum*)	Estrogenic action?	Goat: dry plant 10 g SID [127]	Not reported.	Not reported.

Fennel (*Foeniculum vulgare*)	Estrogenic action?	Not reported	Not reported.	Not reported.

Anise (*Pimpinella anisum*)	Estrogenic action?	Rat: ethanolic and aqueous extract 1 g/kg IP [156]	Not reported.	Not reported.

Goat's rue (*Galega officinalis*)	Estrogenic action?	Sheep: Plant (dry), 2 g/kg body weight in diet daily [158].	Sheep: doses over 5 g/kg were toxic [161].	Not reported.

Asparagus (*Asparagus racemosus*)	Estrogenic action?	Human: concentrated root extract, 60 mg/kg PO SID [166]. Rat: Plant (dry), 2% of the diet [167].	Rat: methanol roots extract in dose of 100 mg/Kg/day for 60 days showed teratological disorders as increased resorption of fetuses, gross malformations as swelling in legs, and intrauterine growth retardation with a small placental size [171].	Not reported.

Milk thistle (*Silybum marianum*)	Estrogenic action?	Bovine: plant (dry), 10 g PO SID [176].	Human: gastrointestinal disturbances have been reported: nausea and diarrhea. Patients who have allergies to other members of the *Asteraceae/Compositae* plant family (including ragweed, marigolds, daisies, etc.) may exhibit allergic reactions to Milk Thistle derivatives [30].	Human: silymarin elimination half-life average 6 hours [30].

Abbreviations: SID, once daily; BID, twice daily; TID, three times daily; PO, oral administration; SC, subcutaneous; IM, intramuscular; IP, intraperitoneal.
